# Biodiesel Production from the Marine Alga *Nannochloropsis oceanica* Grown on Yeast Wastewater and the Effect on Its Biochemical Composition and Gene Expression

**DOI:** 10.3390/plants12162898

**Published:** 2023-08-08

**Authors:** Hoda H. Senousy, Mostafa M. El-Sheekh, Hanan M. Khairy, Heba S. El-Sayed, Ghada Abd-Elmonsef Mahmoud, Amal A. Hamed

**Affiliations:** 1 Botany and Microbiology Department, Faculty of Science, Cairo University, Giza 12613, Egypt; ahamed@sci.cu.edu.eg; 2 Botany Department, Faculty of Science, Tanta University, Tanta 31527, Egypt; mostafaelsheikh@science.tanta.edu.eg; 3 National Institute of Oceanography and Fisheries (NIOF), Cairo 11516, Egypt; hanan_khairy@yahoo.com (H.M.K.); hebasaad2222@yahoo.com (H.S.E.-S.); 4 Botany and Microbiology Department, Faculty of Science, Assuit University, Assuit 71516, Egypt; ghadamoukabel@aun.edu.eg

**Keywords:** *Nannochloropsis oceanica*, yeast wastewater, biodiesel production, N deficiency, N/P ratio, lipid, carbohydrate, Δ9FAD gene expression

## Abstract

Microalgae-based biodiesel synthesis is currently not commercially viable due to the high costs of culture realizations and low lipid yields. The main objective of the current study was to determine the possibility of growing *Nannochloropsis oceanica* on *Saccharomyces cerevisiae* yeast wastewater for biodiesel generation at an economical rate. *N. oceanica* was grown in Guillard F/2 synthetic medium and three dilutions of yeast wastewater (1, 1.25, and 1.5%). Biodiesel properties, in addition to carbohydrate, protein, lipid, dry weight, biomass, lipid productivity, amino acids, and fatty acid methyl ester (FAMEs) content, were analyzed and the quality of the produced biodiesel is assessed. The data revealed the response of *N. oceanica* to nitrogen-deficiency in the three dilutions of yeast wastewater. *N. oceanica* in Y2 (1.25%) yeast wastewater dilution exhibited the highest total carbohydrate and lipid percentages (21.19% and 41.97%, respectively), and the highest lipid productivity (52.46 mg L^−1^ day ^−1^) under nitrogen deficiency in yeast wastewater. The fatty acids profile shows that *N. oceanica* cultivated in Y2 (1.25%) wastewater dilution provides a significant level of TSFA (47.42%) and can be used as a feedstock for biodiesel synthesis. In addition, *N. oceanica* responded to nitrogen shortage in wastewater dilutions by upregulating the gene encoding delta-9 fatty acid desaturase (Δ9FAD). As a result, the oleic and palmitoleic acid levels increased in the fatty acid profile of Y2 yeast wastewater dilution, highlighting the increased activity of Δ9FAD enzyme in transforming stearic acid and palmitic acid into oleic acid and palmitoleic acid. This study proved that the Y2 (1.25%) yeast wastewater dilution can be utilized as a growth medium for improving the quantity of specific fatty acids and lipid productivity in *N. oceanica* that affect biodiesel quality to satisfy global biodiesel requirements.

## 1. Introduction

Fossil fuels are still widely employed in our daily lives despite predictions of energy shortages and the negative effects of their production on the environment. Vital factors behind the encouragement to generate biodiesel from nonfood resources are commercial interest and the growing awareness of environmental and energy challenges. Since algae and halophytes can grow in saltwater and use carbon dioxide (CO_2_) from the air for photosynthesis, they may be viable solutions since they do not require the agricultural land and freshwater needed for the cultivation of agricultural crops [1]. Microalgae may survive in a wide variety of environments. Due to their multicellular structure and adaption mechanism, some species of microalgae are even able to survive in extreme environments [2,3] to produce polysaccharides, proteins, pigments, etc., microalgae are employed in wastewater treatment, biodiesel, and as a promising feedstock in the food, cosmetic industries, pharmaceutical, and feed [4,5,6]. Because of their high lipid content, efficient photosynthesis, and ability to reduce CO_2_ [7], microalgae are considered a promising source for biodiesel production. It has been suggested that oleaginous microalgae species could be useful as a source of feedstock to produce biodiesel [8,9].

Biodiesel cannot be directly generated from microalgae at a commercial scale. Further optimization of growing conditions and an understanding of the process of lipid accumulation is required after developing a high-quality strain of algae [2,10,11]. According to its high lipid productivity, rapid growth, excellent resistance to biotic contamination, ability to meet biodiesel quality standards, high photosynthetic efficiency, and the ability to convert carbon dioxide into lipids in the form of triacylglycerol, *Nannochloropsis oceanica* gained widespread acceptance as a possible candidate for the production of biodiesel [12]. Under optimum growth conditions, they can be used for producing biodiesel by accumulating huge amounts of triacylglycerol (TAGs) [13,14,15].

Microalgae cultivation coupled with wastewater treatment has many benefits, including waste remediation, low-cost microalgal biodiesel generation [16,17], and a high-value end product rich in polyunsaturated fatty acids (PUFAs) [18]. Since massive amounts of water and nutrients can be recycled for algae growth in wastewater-based algal cultivation systems, this integration may offer an economically viable and environmentally beneficial approach for sustainable algal biodiesel generation [19,20,21]. Nitrogen and phosphorus are the two most important nutrients for microalgae, limiting their growth, and overall productivity [22]. Many types of microalgae have been demonstrated to have significant lipid content increases during nutritional stress [23].

Despite the potential benefits of using microalgae, it is not yet feasible to produce them in large quantities due to the high costs associated with the substrates on which they are grown [24]. The cost of producing algal biomass is increased due to the need to use clean water, fertilizers, and CO_2_ injection for various microalgae growing processes, which decreases their attractiveness as technology [25,26]. Municipal wastewater and residual water from agriculture and livestock can be used for cultivation, allowing microalgae to be grown while making use of the nutrients in this type of effluent, reducing some of the drawbacks. By coupling these processes, we can recycle water, produce high-quality effluent, and grow algae for biodiesel [27,28]. Since wastewater commonly has the nutritional, nitrogen, and phosphorus concentrations required for growth, it has been suggested that this type of effluent be used as an alternate substrate for the cultivation of these microorganisms [14,21,24]. By consuming nitrogen and phosphorus from wastewater as lipids and carbohydrates, algae can achieve phytoremediation of wastewater in a more environmentally friendly manner than traditional wastewater treatment processes [29,30].

The identification of several expressed genes that regulate lipid production in microalgae established the way for elucidating the regulation mechanism associated with this process. The regulation mechanism of the lipid biosynthesis of *Dunaliella parva* under nitrogen-deficient circumstances was elucidated by transcriptome sequencing [31,32].

Triacylglycerol is the principal storage lipid in most eukaryotes, particularly microalgae, at the time of environmental stress. Both the acyl-CoA-dependent Kennedy pathway and the acyl-CoA-independent alternative pathway are included in TAG synthesis in algae. Acyl-CoA: diacylglycerol acyltransferases (DGATs) catalyze the last step in TAG biosynthesis via the Kennedy pathway [33]. DGAT1 and DGAT2 are two gene families that code for diacylglycerol acyltransferases. Genes belonging to the DGAT2 family are predominant in the genomes of microalgae [34]. Five DGAT2 genes have been found in *Chlamydomonas reinhardtii* [35]. As reported by Li et al. [33] and Wang et al. [36]. DGAT2 is present in *Nannochloropsis oceanica* eleven times. *Chlorella zofingiensis* possesses eight DGAT2 isoforms, as reported by Mao et al. [37]. Many investigations have found that through environmental stress, algal cells accumulate TAGs but DGAT2 genes show no response at the transcript level. Only one of the five identified DGAT2 genes in *Chlamydomonas reinhardtii* was shown to respond to nitrogen limitation. None of the other four DGAT2 genes showed any significant variation in their transcript levels [35]. Six out of eleven DGAT2 transcripts in *N. oceanica* were found to be upregulated in response to nitrogen deficiency, four copies were found to be upregulated in response to nitrogen sufficiency, and one copy was inactive in response to nitrogen excess, as reported by Li et al. [33]. Nitrogen shortage was shown to increase the expression of four of *Chlorella zofingiensis*’ eight DGAT2 genes [37,38].

In *Nannochloropsis*, 30 percent of the fatty acids stored are in the form of polyunsaturated fatty acids [39]. Most of the lipids’ active and functional properties are due to long-chain polyunsaturated fatty acids (LCPUFAs). Synthesis of LCPUFAs requires multiple types of fatty acid desaturases and elongases, which introduce double bonds and elongate the carbon chain, respectively. Oleic acid (C18:1) and palmitoleic acid (C16:1) are synthesized from stearic acid (C 18:0) and palmitic acid (C16:0) by the enzyme delta 9-desaturase [40].

The objectives of the current research are to evaluate the ability of the *Nannochloropsis oceanica* to grow on low-cost yeast wastewater to replace the F/2 standard enriched seawater medium for reducing production costs. We measured protein, carbohydrate, lipid, amino acid, and fatty acid levels to assess if *N. oceanica* grown in three different dilutions of yeast wastewater (1, 1.25, and 1.5%) exhibited the same biochemical composition as *N. oceanica* grown in the control (F/2) Guillard medium. Additionally, the produced biodiesel’s qualities were evaluated following global criteria concerning data from prior studies.

One of the goals of this study is to verify whether or not a deficiency of nitrogen in yeast wastewater dilutions has any effect on the expression of two crucial enzymes, diacylglycerol acyltransferases (DGAT) and Δ9FAD.

## 2. Results

### 2.1. Evaluation of Dry Weight, Bioactive Components, Biomass, and Lipid Productivity

**Figure 1:**
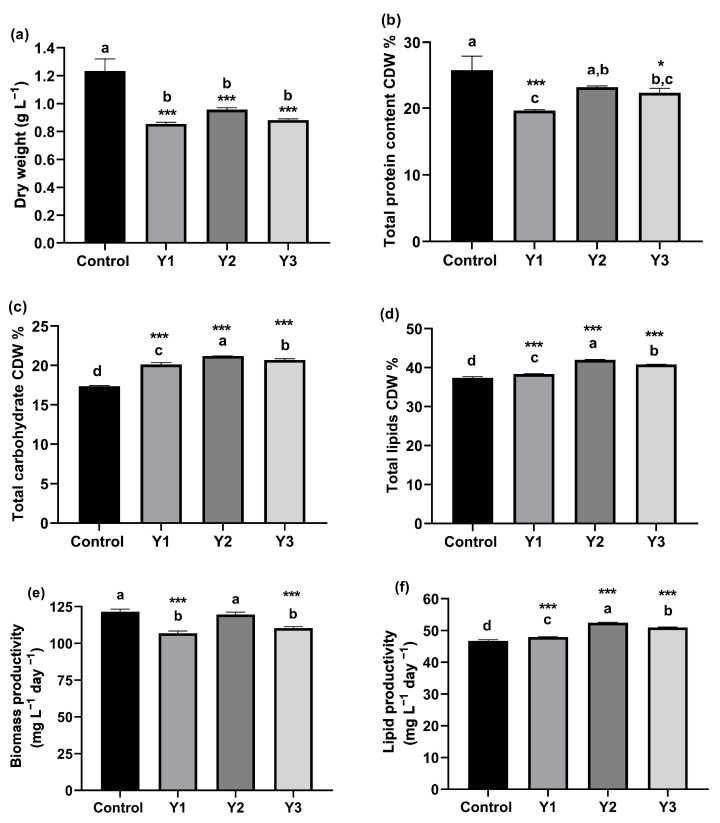
(**a**) The dry weight; (**b**) Total protein; (**c**) Total carbohydrates; (**d**) Total lipids; (**e**) Biomass productivity; (**f**) Lipid productivity of grown *N. oceanica* on three dilutions of yeast wastewater (1, 1.25, and 1.5 %) and control (F/2) for 12 days. * *p* value < 0.05 was considered statistically significant, *** *p* value <0.001 was considered statistically highly significant. Means that do not share a letter are significantly different (Tukey’s test, *p* < 0.05).

Three different dilutions of yeast wastewater (1, 1.25, and 1.5%) were used to cultivate *Nannochloropsis oceanica* at the early stationary phase; after 12 days (late exponential growth phase), samples were collected for biochemical composition analysis. The total protein, lipids, and carbohydrates were analyzed (on a % dry weight basis) in terms of mg/g DW of *N. oceanica*. Additionally, worldwide standards were used to evaluate biodiesel’s properties, and the results were compared to earlier studies. Biochemical changes in *N. oceanica* as a result of yeast wastewater dilutions are illustrated in Figure 1. The biochemical composition of *N. oceanica* was observed to differ significantly (*p* < 0.001) between the three yeast wastewater dilutions (Y1, Y2, and Y3) and the control. The highest cellular dry weight, protein content, and biomass productivity (1.23 ± 0.09 g/L, 25.79 ± 2.11 CDW%, 121.59 ± 1.74 mg/L/day, respectively) were exhibited by the control (F/2) treatment. However, there is no significant difference in protein content and biomass productivity between the control (F/2) and Y2 (1.25%) yeast wastewater dilution. Moreover, the maximum total carbohydrates, and total lipid percentages of dry weight (21.19% ± 0.04 and 41.97% ± 0.13, respectively) were achieved by Y2 (1.25%) yeast wastewater dilution in comparison with other treatments and the control. Furthermore, the Y2 (1.25%) wastewater dilution recorded the highest lipid productivity (52.46 ± 0.16 mg L^−1^ day^−1^).

### 2.2. Amino Acids Estimation

**Figure 2:**
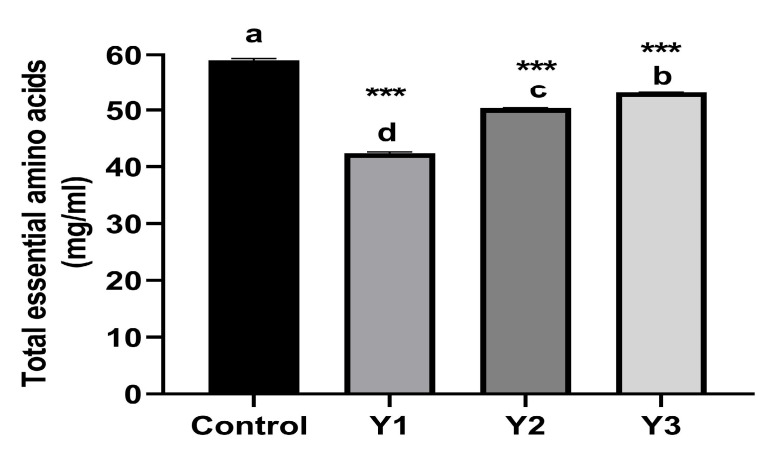

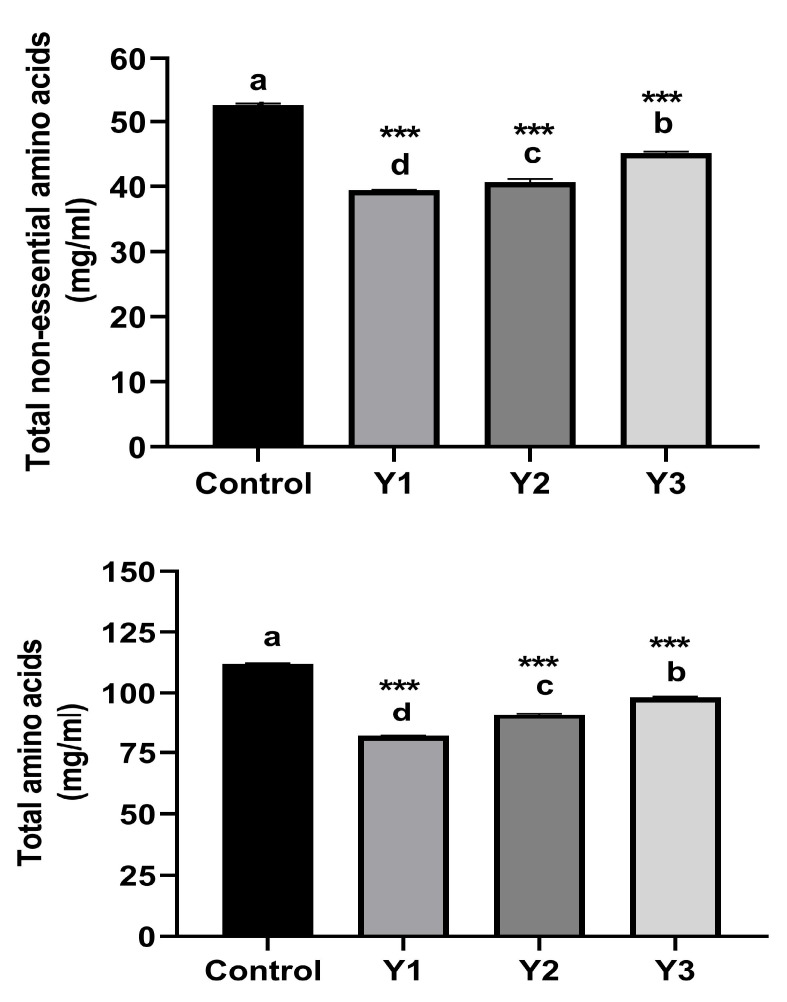
Essential, nonessential, and total amino acids of grown *N. oceanica* on three dilutions of yeast wastewater (1, 1.25, and 1.5%) and control (F/2) for 12 days expressed as mg/mL. *** *p* value <0.001 was considered statistically highly significant. Means that do not share a letter are significantly different (Tukey’s test, *p* < 0.05).

Table 1 and Figure 2 illustrate the amino acid profile of *N. oceanica* grown in 1, 1.25, and 1.5% dilutions of yeast wastewater, as well as the control (F/2). The current investigation found that the amino acid profile did not vary between the different treatments, but the content of specific amino acids, however, varies visibly between the various treatments. The data showed a highly significant difference (*p* < 0.001) in the total amino acids, total essential amino acids, and total nonessential amino acids of *N. oceanica* between the different treatments. The results recorded that *N. oceanica* had the greatest concentrations of total essential amino acids EAA (59.07 ± 0.29 mg/mL), total nonessential amino acids NEAA (52.80 ± 0.26 mg/mL), and total AA (111.87 ± 0.32 mg/mL) at the control (F/2) medium. However, at Y1 (1%) yeast wastewater dilution, the lowest concentrations of total AA, total EAA, and total NEAA were determined. Leucine, lysine, and arginine were found to be the three most prevalent EAA in the control (F/2) medium and the three yeast wastewater dilutions. Meanwhile, the most abundant three NEAA in the control (F/2) medium were alanine, glutamate, and proline. But, glutamate, aspartate, and proline were the three most common NEAA in all three yeast wastewater dilutions.

### 2.3. Fatty Acids Analysis

The fatty acid analysis is widely regarded as a crucial requirement criterion for high-quality biodiesel, where only certain fatty acids can be used for generating biodiesel. Table 2 displays the *N. oceanica* fatty acids profile. According to the results, there was no difference in the profile of fatty acids among the different treatments. Contrary to this, the relative abundance of each fatty acid varies significantly among the treatments. Palmitic acid (C16:0), is the most common saturated fatty acid in all treatments, with its highest value (33.92% ± 0.04) with Y2 (1.25%) yeast wastewater dilution. Stearic acid (C18:0) is the second highest percentage value (5.72% ± 0.11) after Palmitic acid, appearing at Y2 (1.25%) yeast wastewater dilution. Additionally, the most significant value of palmitoleic acid (C16:1) (20.47% ± 0.12) was found in the same dilution (Y2) across all treatments, suggesting it is the most common monounsaturated fatty acid.

**Figure 3:**
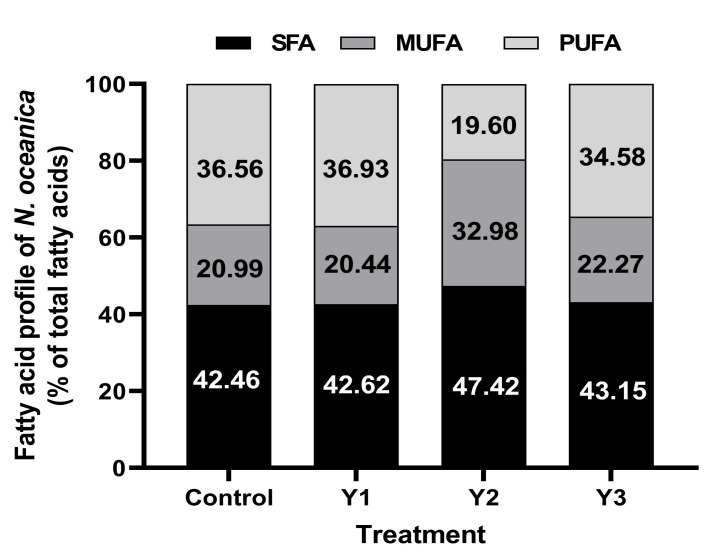
Percentages of total saturated fatty acids (TSFA), total monounsaturated fatty acids (TMUFA), and total polyunsaturated fatty acids (TPUFA) obtained after cultivation of *N. oceanica* at different dilutions of yeast wastewater (1, 1.25, and 1.5%), and control (F/2) for 12 days.

The second monounsaturated fatty acid was oleic acid (C18:1), with the largest percentage value (9.14% ± 0.10) observed with Y2 dilution of yeast wastewater. Furthermore, the findings showed that eicosapentaenoic acid EPA (C20:5) was the most prevalent PUFA with all treatments and the highest value of this fatty acid (3.53% ± 0.04) was obtained with dilution Y2. The second polyunsaturated fatty acid, linoleic acid (C18:2), reached its maximum value (11.95% ± 0.033) at the same dilution (Y2). Thirdly, docosahexaenoic acid DHA (C22:6) was found to have the highest percentage values with dilution Y1, with maximum values of (2.76% ± 0.066), respectively. The most significant levels of linolenic acid (C18:3n6) (0.23% ± 0.014, 0.28% ± 0.022), the fourth polyunsaturated fatty acid, was attained at Y2 (1.25%) yeast wastewater dilution and the control (F/2), respectively. Moreover, the greatest value of linolenic acid (C18:3n3) was detected at Y2 (1.25%) yeast wastewater dilution. It is interesting to note that the majority of common biodiesel fatty acids make up nearly each of these. The data (Figure 3) explained that the highest percentage of TSFA (47.42% ± 0.12) and TMUFA (32.98% ± 0.10) were achieved by Y2 (1.25%) yeast wastewater dilution. However, the greatest percentages of TPUFA (36.93% ± 0.071), and TUSFA (57.54% ± 0.378) were recorded by Y1 (1%) yeast wastewater dilution and the control (F/2), respectively.

### 2.4. Evaluation of Biodiesel Quality

Table 3 shows that the theoretical CN was over the minimum limit of 47 and ranged from 59.94 for Y2 (1.25%) yeast wastewater dilution to 66.65 for Y3 (1.5%) yeast wastewater dilution. The approved range for iodine value according to EN 14214 was (a maximum of 120), where our data recorded that IV ranged between 57.12 and 63.48 for the Y3 (1.5%) and Y2 (1.25%) yeast wastewater dilutions, respectively. Data on CP and PP was supplied concerning ASTM D-6751, −3.12, and −15:10, respectively. ASTM D6751-02 approved viscosity (1.9:6) and ASTM D-6751 range accepted oxidation stability (OS) (minimum 3).

### 2.5. The statistical Evaluation of the Different Parameters

**Figure 4:**
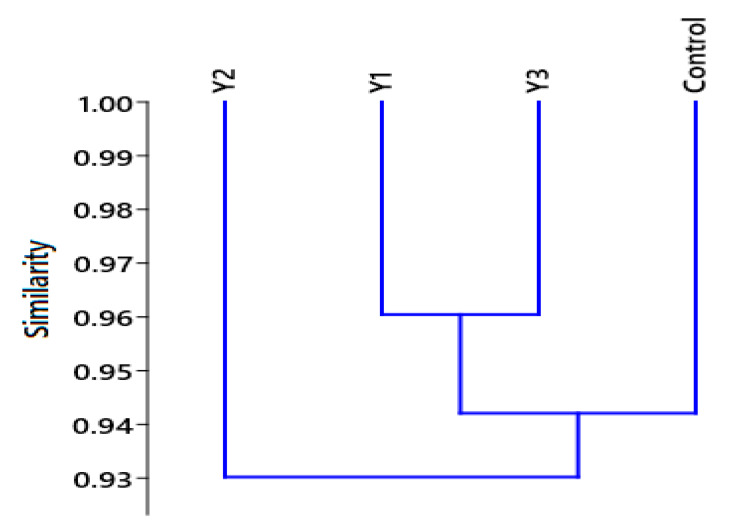
Cluster analysis of different dilutions of yeast wastewater (1, 1.25, and 1.5%), and control (F/2) according to their degree of similarities based on their biochemical constituents and biodiesel properties.

The cluster analysis, Figure 4 showed two main groups; the first one includes Y2 (1.25%) yeast wastewater dilution and the other group includes the control (F/2), Y1 (1%), and Y3 (1.5%) yeast wastewater dilutions with a high degree of similarity between Y1 and Y3.

**Figure 5:**
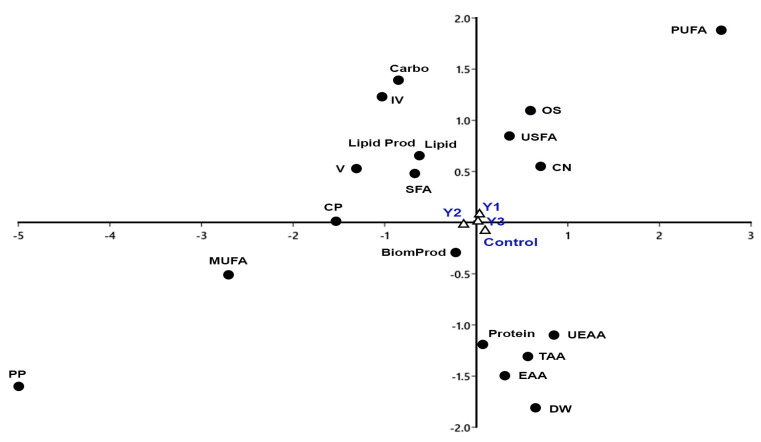
The correspondence analysis (CA) of total protein, carbohydrates, lipids, dry weight, biomass productivity, lipid productivity, fatty acids, and amino acids contents of *N. oceanica*, and biodiesel characters in control and different yeast wastewater dilutions. Protein: total protein, Carbo: total carbohydrates, Lipid: total lipids, DW: dry weight, BiomProd: biomass productivity, LipidProd: lipid productivity, TAA: total amino acids, EAA: essential amino acids, UEAA: nonessential amino acids, SFA: saturated fatty acids, USFA: unsaturated FA, MUFA: Monounsaturated FA, PUFA: Polyunsaturated FA, CN: cetane number, IV: iodine value, CP: Cloud Point, υ: kinematic viscosity and OS: oxidation stability.

The percentage of saturated fatty acids (SFA), as well as total lipids, lipid productivity, total carbohydrates, iodine value (IV), cloud point (CP), and kinematic viscosity (υ), are all strongly correlated with Y2 (1.25%) yeast wastewater dilution, as shown by the correspondence analysis in Figure 5. Polyunsaturated fatty acids (PUFA), unsaturated fatty acids (USFA), cetane number (CN), and oxidation stability (OS) showed a high degree of association with Y1 (1%),and Y3 (1.5%) yeast wastewater dilutions. On the other hand, the control (F/2) treatment had an association with each of the variables dry weight (DW), total amino acids (TAA), essential amino acids (EAA), nonessential amino acids (UEAA), and total protein.

### 2.6. Gene Expression Estimation of Grown N. oceanica on the Yeast Wastewater

**Figure 6:**
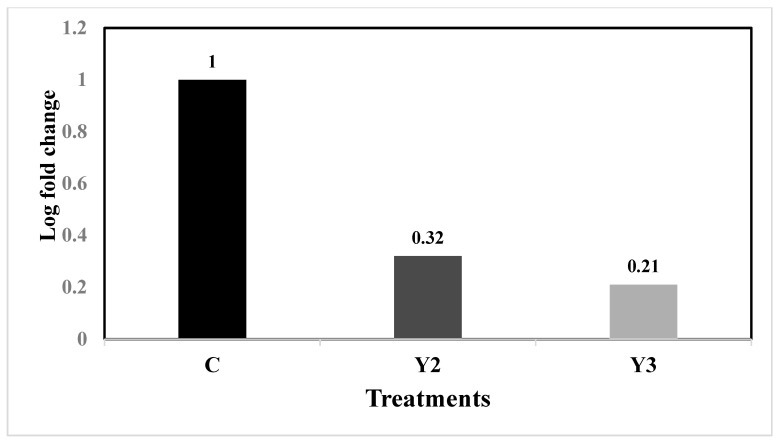
The fold change values for DGAT2G gene under different nitrogen concentrations in the control F/2 (C), Y2 (1.25%), and Y3 (1.5%) yeast wastewater dilutions.

In this investigation, the response of *N. oceanica* to nitrogen-deficient in the three dilutions of yeast wastewater was demonstrated using a molecular genetic study. We applied real-time PCR to examine the relationship between the transcriptional levels of two genes in *N. oceanica* diacylglycerol acyltransferase (DGAT2G) and delta-9 fatty acid desaturase (Δ9FAD) and nitrogen deficiency in the yeast wastewater dilutions based on fold change under different nitrogen concentrations. Only the Y2 (1.25%) and Y3 (1.5%) yeast wastewater dilutions were tested for the abovementioned two genes since these two dilutions recorded the maximum lipid, SFA fatty acid, MUFA fatty acid contents, and the highest activity of the Δ-9 fatty acid desaturase enzyme as shown in Table 3. The activity of the delta-9 fatty acid desaturase (Δ9FAD) enzyme was calculated based on the fatty acid profile provided in Table 3 and the percentages of C16, C18, C16:1, and C18:1. The Y2 and Y3 dilution values for the abovementioned genes were compared with the control (F/2). Reducing nitrogen concentration resulted in a decrease in DGAT2G transcription, as shown in Figure 6. Fold change values for the control, Y2, and Y3 were 1, 0.32, and 0.21, respectively.

**Figure 7:**
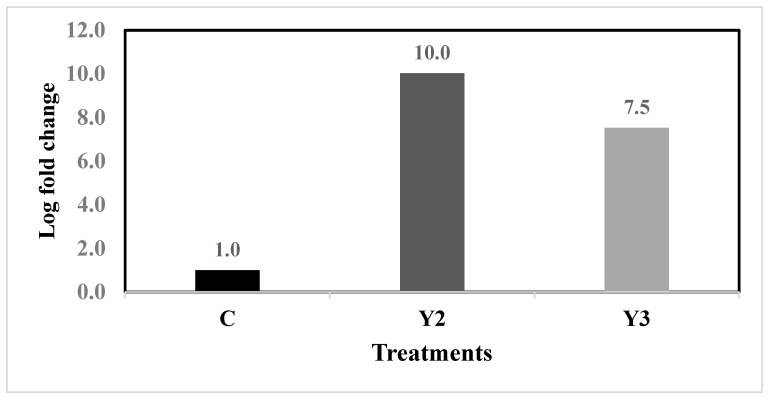
The relation between the fold change of the Δ9FAD gene and the nitrogen deficiency in 1.25% (Y2) and 1.5% (Y3) yeast wastewater dilutions and the control F/2 (C).

Reducing nitrogen content in Y2 and Y3 yeast wastewater dilutions increased delta-9 fatty acid desaturase Δ9FAD expression (Figure 7). Under nitrogen deficiency, the fold change values in Y2 (1.25%) and Y3 (1.5%) wastewater dilutions increased. According to the data, the maximum transcriptional value was achieved for the Y2 (1.25%) yeast wastewater dilution. On the contrary, the fold change values of Y2 and Y3 wastewater dilutions (10, 7.5 respectively) were higher than the control (1.0). The data presented in Table 3 of the fatty acids profile supported these findings when they were used to calculate Δ9FAD (16 + 18) enzyme activity. Based on the results of the calculations, we found that the decreased nitrogen concentration in the yeast wastewater dilutions Y2 and Y3 boosts the enzyme activity. Activity levels for delta-9 desaturase ranged from (42.76%) in Y2 to (34.75%) in Y3 to (31.15%) in the control.

## 3. Discussion

Sustainable energy supply and clean water will be in high demand as the world’s population approaches nine billion in 2050 [41]. Growing energy demands as well as worries about the effects of burning fossil fuels are driving research into renewable energy sources around the world [42]. Microalgae-based biodiesel is being studied by several scientists as a potential alternative to fossil fuels. Biodiesel can be produced from the lipids that microalgae produce [13]. Growing algae for biodiesel production needs to be cost-effective so that the fuel may be used practically. Both nitrogen and phosphorus are essential for algal growth [43]. These nutrients are commonly present in wastewater at concentrations too high for their safe release into the environment, but their removal is costly [44]. Algae cultivation techniques can be updated to increase production while decreasing expenses. Growing algae in wastewater could, therefore, be a cost-effective strategy for wastewater purification and biodiesel production. Strategies for improving the efficiency of biodiesel generation include alternating cultures between nutritional sufficiency and deficiency [45]. Marine microalga *N. oceanica* is commonly grown in Guillard F/2 medium. Currently, the F/2 Guillard medium has several disadvantages due to the different uses of microalgae in biotechnology. Our data revealed that the dilutions of the used yeast wastewater produced biochemical constituents much greater than the F/2 medium (control).

Algal growth and biochemical constituents are greatly influenced by the N/P ratio and the concentration of these elements [46]. The ideal ratio of nitrogen to phosphorus for algal growth was 16:1 [47], although this ratio varied with algal species due to their unique needs for these nutrients. The nitrogen to phosphorus (N/P) ratio in yeast wastewater dilutions is approximately (10:1), compared to the (15:1) seen in the control (F/2) medium. Algae growth was P-limited when the N:P ratio was greater than 14, whereas N-limitation was seen at N:P ratios up to 10 [48].

Previous research [49] has shown that nitrogen deficiency changes the biochemical constituents of microalgae, including their protein, lipid, carbohydrate contents, fatty acids composition, pigments, and photosynthetic suitability. Carbohydrates, lipids, fatty acids contents, and lipid productivity in *N. oceanica* increased due to a reduction of nitrogen in the yeast wastewater dilutions, with the greatest increase observed at Y2 (1.25%) yeast wastewater dilution. However, a drop in dry weight, protein content, biomass productivity, and total amino acids was detected with the reduction in nitrogen level in the yeast wastewater dilutions. On the other hand, the protein level and biomass productivity of the Y2 (1.25%) yeast wastewater dilution are not significantly different from those of the control (F/2). The low (N/P) ratio in yeast wastewater dilutions (10:1) compared to the control (15:1) could result in N limitations in the three dilutions of waste, resulting in an accumulation of carbohydrates, lipids, and fatty acids. Nitrogen deficiency has been shown to have physiological impacts on the macroalga *Gracilariopsis lemaneiformis* in recent work by Liu et al. [50], where the measurements of physiological parameters revealed that under nitrogen stress, amino acids, and protein production were reduced while soluble polysaccharide levels were elevated, thus our findings are supported by these results. Our results agree with those of other researchers who found that low nitrogen levels decrease protein synthesis necessary for cell division and photosynthesis, slowing growth rates and, ultimately, biomass production [51,52]. Similarly, to our results, Millán-Oropeza et al. [53] found that *Chlorella* sp. accumulated carbohydrates in response to nitrogen deficiency. Algae store organic carbon as starch and lipids in cases of nitrogen-limited supplies, offering algal biomass an attractive feedstock for biodiesel synthesis [54,55,56].

Microalgae metabolic pathways may be altered toward lipids production, and triglyceride accumulation if nutrients are depleted due to wastewater dilution [26,41]. Nitrogen shortage has been linked to lipid accumulation in earlier studies [26,41,57]. Changes in the SFA values in *Dunaliella tertiolecta* were correlated closely with variations in nitrogen availability [26]; therefore, these findings are consistent with our findings. Biodiesel can be produced from *N. oceanica* grown in yeast wastewater due to the high palmitic acid (C16:0) level (29.24% ± 0.11) at Y2 (1.25%) yeast wastewater dilution. According to our results, palmitic acid was the most common SFA in all treatments with its highest level at Y2 (1.25%) yeast wastewater dilution, and this finding supported the findings of Abugrara et al. [58] who found palmitic acid to be an essential part in the basic lipids of *N. oceanica*. According to our results, SFA was more prevalent in *N. oceanica* than MUFA and PUFA for all treatments, Guihéneuf and Stengel [59] suggested this result. In addition, the dilution Y2 (1.25%) yielded the highest values of SFA, MUFA, and PUFA relative to the control, which may be due to the reduction of nitrogen in yeast wastewater dilutions. There is evidence that lowering the nitrogen content of the culture medium increases the percentages of SFA, MUFA, and PUFA [60,61,62]. Most algae are rich in polyunsaturated fatty acids (PUFAs), which are among the most nutritionally significant and essential fatty acids since they are crucial nutrients in animal nutrition [63].

Fe, Mn, Mo, Co, Na, Zn, and EDTA were all detected in the yeast wastewater but at lower concentrations than in the Guillard F/2 medium (control). However, the yeast wastewater displayed lower concentrations of N and P than the Guillard F/2 medium (control). Yeast wastewater used in this study is an ideal environment for *N. oceanica* growth according to Darki et al. [62] who mentioned that a medium rich in sodium and trace elements including Fe, Mn, Mo, and Co but deficient in N and P proved optimal for algal growth [62]. These nutrients had a positive association with both high total lipid levels and high concentrations of saturated fatty acids, especially palmitic acid. Moreover, the incorporation of Fe^3+^, Zn^2+^, Mn^2+^, Mo^6+^, and EDTA into the culture medium has been shown to boost lipid productivity [64].

Since most biodiesel is composed of fatty acids with carbon numbers between 16 and 18 [65], it is important to estimate the chemical constituents of *N. oceanica* before using it as a biodiesel source. Our biodiesel is primarily composed of C16-C18 fatty acid esters (stearic acid, palmitic acid, linoleic acid, oleic acid, and linolenic acid). The growth of *N. oceanica* on the different dilutions of the low-cost yeast wastewater improves biodiesel efficiency qualities due to the increased FAME content [66]. The most crucial characteristics of biodiesel are its cloud point (CP), viscosity (υ), cetane number (CN), pour point (PP), iodine value (IV), and oxidation stability (OS), as established by ASTM D-6751 and EN 14214 [67,68]. Data from this study were compared to ASTMD-6751 or EN 14214 standards for biodiesel and found to be within an acceptable range for kinematic viscosity (υ), oxidation stability (OS), cloud point (CP), cetane number (CN), pour point (PP), and iodine value (IV) [69]. The minimum CN for biodiesel depending on the ASTM D6751-02 standard is 47.0, whereas the maximum IV is 120 g I_2_/100 g fat. Fatty acid methyl ester composition has been used for calculating the CN number and IV [70]. Indirect evaluations of oxidative biodiesel stability can be made using linoleate (C18:2) and linolenate (C18:3) [29]. Fuels with plenty of SFA benefit from the CN since it makes them more ignited. The presence or absence of double bonds (saturated or unsaturated) and the proportion of long- to short-chain fatty acids in the biodiesel’s constituents both determine the fuel’s quality [69]. In general, a longer chain length indicates a higher quality fatty acid (C16-C18) concerning combustion heat, viscosity, and cetane number (CN) [71]. Standard cetane number (CN) conformity is required for high-quality biodiesel since this number reflects ignition quality, pollution levels, density, and viscosity [72].

One of the Kennedy fatty synthesis pathways is diacylglycerol acyltransferase (DGAT). The final step in the production of triacylglycerol is activated by acyl-CoA: diacylglycerol acyltransferase (DGAT) [38]. Both DGAT-1 (including DGAT-1A, and DGAT-1B) and DGAT-2 (containing 12 isoforms [36,73] have been identified in *Nannochloropsis* spp. [34]. The expression of DGAT2G was suppressed in the present research when nitrogen levels were low. This finding is consistent with previous studies showing that N shortage upregulates some types of DGAT2 while downregulating others, such as DGAT2G [33,74].

In *N. oceanica*, 6 DGAT2 encoding genes were increased in response to N deprivation, while the remaining DGAT2 encoding genes were downregulated. That was discovered by Zienkiewicz et al. [75]. Similar results have been reported in *Chlamydomonas*, where there are five DGAT-encoding genes in the genome but only one is upregulated in the presence of a nitrogen deficiency [35,76]. Zienkiewicz et al. [75] explain the variation in DGAT gene expression by observing that different DGAT encoding genes respond differently to environmental stress, including certain genes involved in TAG synthesis during nitrogen depletion and others during nitrogen repletion.

Several investigations have looked for evidence that desaturase gene expression correlates with changes in fatty acid content during nitrogen shortage. In the initial step of fatty acid desaturation, 9-desaturase forms a double bond at the Δ9 position and is, thus, the most important desaturase enzyme [77,78,79]. Stearic acid (C18:0) and palmitic acid (C16:0) are converted to oleic acid (C18:1) and palmitoleic acid (C16:1) by this enzyme [40,80]. The present data showed that palmitoleic and oleic acid levels are increased under nitrogen deficiency especially in the Y2 (1.25%) yeast wastewater dilution. Liang et al. [74] and Meng et al. [81] findings are consistent with our findings. Under nitrogen depletion conditions, Δ9 desaturase expression was shown to be elevated in the current investigation. The upregulation of fatty acid desaturase (Δ9FAD) gene expression in *Auxenochlorella pyrenoidosa* in response to nitrogen shortage is consistent with this finding Zhang et al. [82]. The transcripts of delta-9 fatty acid desaturase were increased in *Chlorella zofingiensis* in response to abiotic stresses such as nitrogen deficiency and intense light [83]. The Δ9 fatty acid desaturase in *Xanthonema hormidioides* was shown to be increased in a proteomic investigation performed by Gao et al. [79] when the organism was exposed to low nitrogen and low temperature. Increased reactive oxygen species (ROS) under extreme conditions, such as nitrogen deficiency, leads to upregulation of genes involved in fatty acid production and the buildup of fatty acids and desaturated fatty acids [33,83]. Research has shown that these fatty acids can decrease ROS and preserve cells from harm.

## 4. Materials and Methods

### 4.1. Algal Strain and Culture Conditions

**Figure 8:**
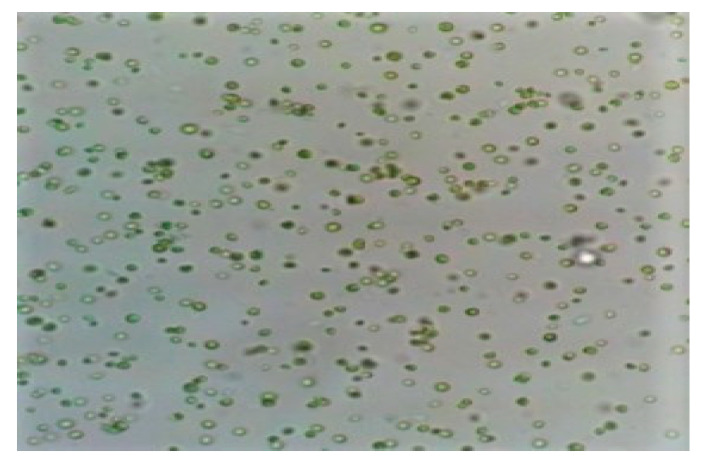
*Nannochloropsis oceanica* viewed under a light microscope.

The algal unit at the marine hatchery at Egypt’s National Institute of Oceanography and Fisheries has generously provided *Nannochloropsis oceanica* displayed in Figure 8.

Stock culture of *N. oceanica* was cultivated on F/2 enriched seawater medium [84] in 250 mL flasks containing 100 mL sterilized culture medium. The F/2 medium containing 75 g/L NaNO_3_, 5 g/L NaH_2_PO_4_·2H_2_O, 1 mL of stock trace metal solution (per 1 L seawater, 3.15 g FeCl_3_·6H_2_O, 1 mL 22.0 g/L ZnSO_4_·7H_2_O, 1 mL 9.8 g/L CuSO_4_·5H_2_O, 1 mL 6.3 g/L Na_2_MoO_4_·2H_2_O, 1 mL 180.0 g/L MnCl_2_·4H_2_O, and 1 mL 10.0 g/L CoCl_2_·6H_2_O), and 0.5 mL of stock vitamin solution (per 1 L, 200 mg thiamine HCl (vitamin B1), 1 mL 1.0 g/L cyanocobalamin (vitamin B12), and 1 mL 1.0 g/L biotin (vitamin H), all in seawater. Algae were cultivated in a static incubator at 25 ± 2 g/L salinity, pH 7.8, and temperature 21 ± 2 °C with a regular airflow. They were illuminated with 80 μmol photons m^2^ s^1^ of cool-white fluorescent for 24 h.

### 4.2. Waste Effluent

The studied yeast wastewater was obtained from an industrial yeast production factory (The Egyptian Starch, Yeast, and Detergents Company, Alexandria, Egypt) from the outlet stream. The raw yeast wastewater was pretreated by centrifuging it for 15 min at 4000 rpm to remove particles and then autoclaving it to kill any remaining yeast or bacteria. This was performed so that only waste components for algal utilization were used. Chemical analysis [85] of the waste is displayed in Table 4 below. In order to save money concerning the high-cost components of the F2 medium, sterilized yeast wastewater was mixed with filtered seawater at varying dilutions in the medium preparation for the experiment.

### 4.3. Experimental Design

**Figure 9:**
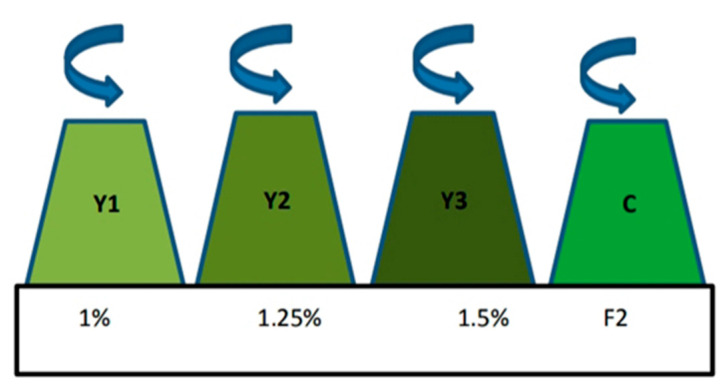
Visual representation of the experimental setup.

Initial experiments with yeast wastewater dilutions (1, 1.25, 1.5, 1.75, and 2.0%) for growing *N. oceanica* showed that (1, 1.25, and 1.5% represented by Y1, Y2, and Y3, respectively, in triplicates) were the best for algal growth when compared to F/2 synthetic medium (control) as shown in Figure 9. Biochemical analysis of the algal cells was performed after 12 days (late exponential growth phase).

### 4.4. Algal Growth and Biomass Assay

Cell dry weight (CDW) was used as an indicator of algal growth and expressed as g/L.

### 4.5. Biomass Productivity

Abomohra et al. [86] utilized the following equation to determine biomass productivity:

Biomass productivity (g CDW L^−1^ d^−1^) = (CDW_L_ − CDW_E_) · (T_L_ − T_E_)^−1^

with CDWE representing the CDW (g L^−1^) at days of early exponential phase (t_E_) and CDWL at days of late exponential phase (t_L_).

### 4.6. Biochemical Analysis

#### 4.6.1. Protein

The colorimetric Lowry approach [87] was used for determining the estimated concentrations of protein. Bovine serum albumin was employed as a standard. Algal culture (1 mL) was hydrolyzed in 1 M NaOH solution (10 mL) for 24 h, according to the protocol. The centrifuged supernatant was analyzed for the amount of protein. A total of 5 mL of reagent “A” were added to 1 mL of the hydrolysate supernatant and thoroughly mixed for 10 min at room temperature. Reagent “B” (1 mL) was added, shaken gently, and left to incubate for 30 min at room temperature. The mixture’s absorbance was evaluated at a wavelength of 750 nm.

#### 4.6.2. Carbohydrate

Dubois’ colorimetric phenol–sulfuric acid method [88] was used to determine the total carbohydrate content. Biomass (10 mg) was dissolved in water (10 mL) to create a standard sample concentration (1 mg mL^−1^). In a water bath, one milliliter of samples was exposed to three milliliters of concentrated sulfuric acid (72 wt%) and one milliliter of phenol (5%, *w*/*v*). For 5 min, the resulting mixtures were heated to a temperature of 90 °C. The spectrometer’s absorbance at 490 nm was then recorded. The absorbance readings were then compared to a glucose standard curve.

#### 4.6.3. Lipid Extraction and Methylation

The Bligh and Dyer [89] technique was used for measuring total lipids. The samples were homogenized in a mixture of chloroform and methanol (1:2) and left to settle in the dark for 12 h. Chloroform and methanol were used in very low concentrations to extract the residues multiple times. To evaporate the chloroform layer, it was first extracted using a separating funnel. Weighting the residues allowed us to determine their lipid content, which we then expressed as a percentage of their dry weight. Esterified fatty acids (EFA) from intracellular lipid extracts were transmethylated for GC analysis, as previously described by [90,91]. The fatty acid methyl esters were analyzed using GC and GC/MS. The GC analysis was carried out using Device Model: HP (Hewlett Packard) 6890 GC, using a capillary column HP-INNO Wax (Polyethylene glycol), 0.25 mm ID, 60 m, 0.2 µm film thickness. Detector temperature: 250 °C. Injector temperature: 220 °C. Injection volume: 3 µL, split ratio 50:1.

### 4.7. Lipid Productivity

The lipid productivity was calculated according to Andrade and Costa [92], and modified by Abomohra et al. [86]:

Lipid productivity (mg L^−1^ d^−1^) = (TL_L_ − TL_E_) · (t_L_ − t_E_)^−1^

where TL_E_ represents the lipid content during the early exponential phase (t_E_), while TL_L_ represents the total lipid content during the late exponential phase (t_L_).

### 4.8. Amino Acids

Except for tryptophan, each amino acid was extracted and measured with a Beckman 119 CL amino acid analyzer, as described by Spackman et al. [93]. The reaction of the amino acid analyzer was evaluated by measuring the concentration of a standard mixture of seventeen naturally occurring amino acids in protein hydrolysate.

### 4.9. Produced Biodiesel Theoretical Features

Using GC analysis for determining fatty acid profiles, the Biodiesel Analyzer evaluates the resulting biodiesel’s qualities (version 2.2 (2016) BiodieselAnalyzer© Version 2.2. Release Date: 1 February 2016 or http://www.brteam.ir/analysis/ (accessed on 31 July 2023)) [69,94,95].

### 4.10. RNA Extraction

Samples were collected from control and nitrogen depletion treatments (Y2 and Y3) after 12 days. In liquid nitrogen, samples were ground to extract total RNA. According to (Gene DireX kit, Taipei, Taiwan) mRNA was extracted. mRNA was reversely transcribed to synthesize the first DNA strand (cDNA) by using the Xpert cDNA super mix system (GRiSP research solutions, Porto, Portugal).

### 4.11. RTq PCR

Xpert fast Sybr Green Master Mix with ROX (Grisp research, Portugal) was used for qPCR reaction. Real-time PCR amplification was performed using BRO8301 (TaKaRa, Shiga, Japan). Duplicates were used for each sample (control, Y2, and Y3) in the RT-PCR reaction. Specific primers for real-time PCR were designed to detect mRNA levels of DGAT2G and delta-9 fatty desaturase genes from different samples. The Actin gene served as a reference gene. Primers used in RT-PCR were synthesized by Eurofins Genomic, Germany.

The RTq PCR condition was as follows: first, denaturation at 95 °C for 2 min; 40 cycles which comprised denaturation, annealing, and extension at temperatures (95, 59, and 72 °C) for 30 s, respectively; and then, final extension stage at 72 for 3 min. Ct values were determinate to each duplicate for each sample. Relative gene expression was calculated using the 2^−ΔΔCt^ method [96], and using actin amplification product as the internal standard. The amplification products sizes were 160–200 bp. Primers were designed by using the Blast program and from NCBI published data (Table 5).

### 4.12. Enzyme Activity

Okada et al. [97] used the following equation to determine the activity of the delta-9 desaturase enzyme:

Δ9 − desaturase (16 + 18) = [(C16:1 + C18:1)/(C16:1 + C16:0 + C18:1 + C18:0)] × 100.

### 4.13. Statistical Analysis

Data represented as mean of three replicates ± SD. The normality of the data was tested using the Kolmogorov–Smirnov test. The significant variations between groups of treatments were identified using a one-way analysis of variance (ANOVA). All groups were compared to the control group using the Dunnet test to demonstrate the treatment’s significance. Tukey post hoc comparisons among different groups were performed. Statistically significance difference was checked at <0.05 (*p*-value). IBM SPSS Statistics for Windows Version 27, GraphPad Prism Version 8, and Microsoft Excel 365 were used to analyze the data. Cluster analysis correspondence analysis (CA) and principal component analysis (PCA) were performed using PAST, V4.08 [98].

## 5. Conclusions

In summary, the study results reveal that yeast wastewater was successfully used for culturing N. oceanica, as the limiting nitrogen source, leading to a cost-effective and potential alternative commercial medium for biomass production without the need for costly carbon sources in the cultivation medium. Since N. oceanica can grow in wastewater without added nutrients, this would result in significant benefits. To increase lipid productivity, and the amount of specific fatty acids in N. oceanica that affect biodiesel standards according to international biodiesel criteria, this study displayed that the Y2 (1.25%) yeast wastewater dilution can be used as a growth medium instead of Guillard F/2 synthetic medium. Also, the percentages of monounsaturated fatty acids (palmitoleic acid and oleic acid) elevated significantly, and neutral lipid composition and fatty acid distribution were altered. The high oleic acid percentage supplies numerous benefits, including enhanced oxidative stability and a broad temperature range of use. N. oceanica grown in Y2 yeast wastewater dilution has elevated levels of TSFA and TMUFA, and the oil that it produces has an elevated percentage of 16–18 carbon chain fatty acids, thus serving as a promising candidate to be utilized as biodiesel. Therefore, N. oceanica is suitable as a feedstock in the production of biodiesel. In response to the shortage of nitrogen in yeast wastewater dilutions, the Δ9FAD gene was upregulated, particularly the Y2 dilution. We need to conduct studies with N. oceanica in the future to determine the optimal growth conditions for enhanced lipid production, such as changing the temperature and light intensity. Altering the Δ9FAD gene in oleaginous N. oceanica will also be necessary to increase lipid production with a sufficient amount of oleic acid for the generation of higher-quality biodiesel. Also, nitrogen limitation and the various isoforms of the DGAT genes in N. oceanica need more research.

## Figures and Tables

**Table 1:** Total amino acids of grown *Nannochloropsis oceanica* on three dilutions of yeast wastewater (1, 1.25, and 1.5%) and control (F/2) for 12 days expressed as mg/mL.

Amino Acids	Control	Y1	Y2	Y3
Essential AA (mg/mL)				
Arginine	6.74 ± 0.03	5.32 ± 0.05	5.93 ± 0.05	6.14 ± 0.05
Histidine	4.52 ± 0.04	2.43 ± 0.04	2.9 ± 0.02	3.27 ± 0.06
Isoleucine	6.17 ± 0.04	4.07 ± 0.04	5.67 ± 0.07	5.86 ± 0.07
Leucine	7.36 ± 0.08	5.93 ± 0.07	6.5 ± 0.02	6.23 ± 0.04
Lysine	6.78 ± 0.07	6.29 ± 0.05	9.32 ± 0.03	7.76 ± 0.08
Methionine	5.91 ± 0.04	3.29 ± 0.04	4.3 ± 0.03	4.18 ± 0.04
Phenylalanine	5.67 ± 0.07	4.19 ± 0.03	4.33 ± 0.03	5.28 ± 0.06
Threonine	4.83 ± 0.09	3.7 ± 0.04	3.78 ± 0.03	4.89 ± 0.06
Tryptophan	5.82 ± 0.05	3.32 ± 0.06	2.03 ± 0.06	4.75 ± 0.11
Valine	5.27 ± 0.05	3.84 ± 0.07	5.53 ± 0.09	4.72 ± 0.05
Total EAA (mg/mL)	59.07 ± 0.29 ^a^	42.39 ± 0.21 ^d^	50.30 ± 0.05 ^c^	53.07 ± 0.07 ^b^
Nonessential (mg/mL)				
Alanine	10.40 ± 0.15	4.68 ± 0.05	5.45 ± 0.18	5.16 ± 0.07
Aspartate	5.62 ± 0.07	7.28 ± 0.03	6.23 ± 0.12	8.18 ± 0.05
Cysteine	4.19 ± 0.03	2.8 ± 0.02	4.4 ± 0.07	3.47 ± 0.04
Glutamate	10.21 ± 0.05	8.62 ± 0.06	6.62 ± 0.06	9.17 ± 0.05
Glycine	5.53 ± 0.06	3.61 ± 0.07	4.42 ± 0.09	4.72 ± 0.06
Proline	9.45 ± 0.08	6.36 ± 0.05	6.28 ± 0.06	7.47 ± 0.14
Serine	4.85 ± 0.08	3.83 ± 0.09	5.04 ± 0.04	4.64 ± 0.05
Tyrosine	2.56 ± 0.05	2.26 ± 0.11	2.22 ± 0.09	2.33 ± 0.07
Total NEAA	52.80 ± 0.26 ^a^	39.45 ± 0.09 ^d^	40.66 ± 0.5 ^c^	45.15 ± 0.22 ^b^
Total AA	111.87 ± 0.32 ^a^	81.84 ± 0.13 ^d^	90.96 ± 0.5 ^c^	98.22 ± 0.24 ^b^

The means that do not share a letter are significantly different. (Tukey Pairwise Comparisons. *p* < 0.05.)

**Table 2:** The fatty acids profile of grown *N. oceanica* on three dilutions of yeast wastewater (1, 1.25, and 1.5%), and control (F/2) for 12 days was estimated as a proportion of the whole fatty acid content.

Fatty Acid	C	Y1	Y2	Y3
C14:0	1.99 ^c^ ± 0.094	2.14 ^c^ ± 0.055	2.92 ^a^ ± 0.052	2.40 ^b^ ± 0.116
C15:0	0.71 ^b^ ± 0.068	0.79 ^ab^ ± 0.047	0.86 ^a^ ± 0.004	0.82 ^a^ ± 0.013
C16:0	31.81 ^b^ ± 0.156	31.58 ^b^ ± 0.039	33.92 ^a^ ± 0.04	31.19 ^b^ ± 0.165
C17:0	0.40 ^b^ ± 0.022	0.58 ^a^ ± 0.011	0.62 ^a^ ± 0.018	0.63 ^a^ ± 0.071
C18:0	4.78 ^b^ ± 0.048	4.43 ^c^ ± 0.107	5.72 ^a^ ± 0.11	4.77 ^b^ ± 0.017
C21:0	0.90 ^b^ ± 0.007	1.47 ^a^ ± 0.123	1.53 ^a^ ± 0.060	1.50 ^a^ ± 0.038
C24:0	1.86 ^a^ ± 0.100	1.63 ^b^ ± 0.007	1.83 ^a^ ± 0.053	1.84 ^a^ ± 0.049
% of TSFA	42.45 ^b^ ± 0.378	42.62 ^b^ ± 0.118	47.42 ^a^ ± 0.12	43.15 ^b^ ± 0.212
C14:1	0.18 ^a^ ± 0.009	0.17 ^ab^ ± 0.005	0.14 ^c^ ± 0.006	0.16 ^bc^ ± 0.010
C15:1	0.10 ^a^ ± 0.007	0.08 ^b^ ± 0.009	0.07 ^c^ ± 0.003	0.08 ^b^ ± 0.002
C16:1	13.43 ^b^ ± 0.095	13.26 ^b^ ± 0.117	20.47 ^a^ ± 0.12	13.97 ^b^ ± 0.101
C17:1	0.61 ^a^ ± 0.087	0.51 ^b^ ± 0.070	0.59 ^a^ ± 0.041	0.53 ^b^ ± 0.055
C18:1n9	3.13 ^c^ ± 0.106	4.26 ^b^ ± 0.073	9.14 ^a^ ± 0.10	5.18 ^b^ ± 0.047
C20:1	2.81 ^a^ ± 0.151	1.58 ^c^ ± 0.068	1.78 ^b^ ± 0.042	1.57 ^c^ ± 0.053
C22:1	0.72 ^a^ ± 0.068	0.57 ^b^ ± 0.068	0.78 ^a^ ± 0.050	0.78 ^a^ ± 0.064
% of TMUFA	20.99 ^c^ ± 0.248	20.44 ^d^ ± 0.070	32.98 ^a^ ± 0.10	22.27 ^b^ ± 0.184
C18:2n6	13.52 ^a^ ± 0.028	13.50 ^a^ ± 0.134	11.95 ^b^ ± 0.033	11.61 ^c^ ± 0.023
C20:2n6	1.01 ^a^ ± 0.016	0.61 ^c^ ± 0.031	0.69 ^b^ ± 0.034	0.66 ^b^ ± 0.006
C18:3n6	0.28 ^a^ ± 0.022	0.14 ^c^ ± 0.066	0.23 ^ab^ ± 0.014	0.19 ^bc^ ± 0.013
C18:3n3	1.73 ^a^ ± 0.089	1.46 ^b^ ± 0.049	1.86 ^a^ ± 0.126	1.83 ^a^ ± 0.087
C20:5n-3	17.85 ^b^ ± 0.148	18.46 ^a^ ± 0.035	3.53 ^c^ ± 0.04	17.99 ^b^ ± 0.013
C22:6n-3	2.17 ^b^ ± 0.154	2.76 ^a^ ± 0.066	1.29 ^c^ ± 0.10	2.30 ^b^ ± 0.110
% of TPUFA	36.56 ^b^ ± 0.197	36.93 ^a^ ± 0.071	19.60 ^c^ ± 0.10	34.58 ^b^ ± 0.042
Total TUSFA	57.54 ^a^ ± 0.378	57.38 ^a^ ± 0.118	52.57 ^b^ ± 0.12	56.85 ^a^ ± 0.212
Δ9FAD (16 + 18)	31.15 ^d^ ± 0.305	32.74 ^c^ ± 0.179	42.76 ^a^ ± 0.180	34.75 ^b^ ± 0.290

SFA; saturated fatty acids, USFA; unsaturated FA; MUFA Monounsaturated FA; PUFA Polyunsaturated FA. The means that do not share a letter are significantly different. Tukey Pairwise Comparisons. *p* < 0.05. Means ± SD, *n* = 3.

**Table 3:** Biodiesel Analyzed software version 2.2 in 2016 was used to evaluate the theoretical biodiesel qualities of the gained fatty acids for *N. oceanica* under three dilutions of yeast wastewater (1%, 1.25, and 1.5%) and control (F/2).

Properties	Control	Y1	Y2	Y3	Accepted Range	Standard Ref.
CN_min_	66.27	66.12	59.94	66.65	47	ASTM D6751-02
IV_max_	58.58	59.99	63.48	57.12	120	EN 14214
CP_min_	11.74	11.62	12.89	11.41	−3.12	ASTM D-6751
PP	5.92	5.79	7.17	5.57	−15:10	ASTM D-6751
υ_mm^2^/s_	2.79	2.76	3.35	2.78	1.9:6	ASTM D6751-02
OS_min_	10.18	10.40	10.99	11.24	3	ASTM D-6751

Biodiesel Analyzed software version 2.2 (2016). Cetane Number (CN), Iodine Value (IV), Cloud Point (°C) (CP), Pour Point (°C) (PP), Kinematic Viscosity (mm^2^/s) (υ), and Oxidation Stability (h) (OS).

**Table 4:** Mineral constituents of the yeast wastewater.

Mineral Constituents	g/L	Mineral Constituents	g/L
Ca(NO_3_)_2_	0.03	MnSO_4_	0.50
CaCl_2_	0.03	Na_2_EDTA	2.40
CoCl_2_	4.70	Na_2_MoO_4_	1.48
CuSO_4_	9.80	NaH_2_PO_4_	3.66
FeCl_3_	1.77	NaNO_3_	36.60
KNO_3_	0.03	NiCl_2_	1.49
MgSO_4_	0.04	ZnCl_2_	1.50
MnCl_2_	9.10	ZnSO_4_	0.10

**Table 5:** Sequences of the primers used in RTq PCR.

Gene Name	Gene Accession Number	^5′^ Forward Primer ^3′^	^5′^ Reverse Primer ^3′^
Actin	XM_005852283	ATGGTGGGGATGGACCAGAA	CTCCGTGAGAAGAACGGGAT
Diacylglycerol acyltransferase DGAT2G	>KX867962.1	CGAGGCTTCCCTTGAGCAAT	AGGCATTCAAAACGACTGCTG
Delta 9 desaturase	>KY214449.1	TTCTGGAACGCCTTTTGGGT	CCTTCTCCGATCGCCACTAC

## Data Availability

The data presented in this study are available upon request from the corresponding author.
